# Supplementary material for: Molecular evidence supports a genic capture resolution of the lek paradox
Source: Nat Commun. 2019 Mar 25;10:1359. doi: 10.1038/s41467-019-09371-y (PMC6433924; doi:10.1038/s41467-019-09371-y)
Supplement: Supplementary file 1 — Supplementary Information [file 41467_2019_9371_MOESM1_ESM.pdf]

Molecular evidence supports a genic capture resolution of the lek paradox

Dugand et al.

Supplementary Discussion

Our results suggest that sexual selection purged variation in the success-selected lines. However, the effects of genetic drift are enhanced in small populations and a similar pattern of reduced genetic diversity could also be explained by lower N_e in the success-selected lines. Due to the larger fluctuations in allele frequencies, rare alleles will more readily be lost in small populations, potentially generating the consistent pattern of purging that we see in our success-selected lines. F_{ST} among success-selected lines was higher than among failure-selected lines (0.182 and 0.162, respectively), consistent with the hypothesis that the N_e of success-selected lines was lower. Our experimental design controlled the number of breeding individuals, but did not directly control N_e. We evaluated the effect of the variation in N_e on molecular genetic variation using simulations (see Methods).

The results from the simulations are shown in Supplementary Figures 2 and 3. As expected, $\mathbf{P_{S<F}}$ increased with $\mathbf{D}\boldsymbol{F}_{\mathbf{ST}}$, revealing that increased genetic drift causes rare variants to become fixed more readily and consistently in smaller populations. However, our observed $\mathbf{P_{S<F}}$ value (for the 1,363 positive *DiffStats*; shown in blue) falls well outside the 95% confidence intervals expected by chance. Therefore, while it is possible to generate a similar $\mathbf{P_{S<F}}$ value through drift, the associated $\mathbf{D}\boldsymbol{F}_{\mathbf{ST}}$ value needs to be substantially larger than our observed value for drift alone to explain the pattern. A similar pattern emerges for the genome-wide $\mathbf{D}\boldsymbol{H}_{\mathbf{e}}$ estimate, with our observed value of $\mathbf{D}\boldsymbol{H}_{\mathbf{e}}$ falling outside of the 95% confidence intervals (Supplementary Figure 2).

Changing $N_{failure}$ from 50 to 33 increased the amount of genetic drift among failure-selected lines. By comparing Supplementary Figures 2 and 3, we are able to test whether different absolute N_e (i.e. not just $N_{failure} = 50$) could generate our observed results; our simulations suggest not. Importantly, F_{ST} values from the simulations (~0.22 when $N_e = 33$ and ~0.16 when

N_e = 50) were similar to our observed values, and suggest that the N_e of our experimental lines were in the range of ~33-42 individuals. Therefore, these simulations seem to accurately reflect our data.

In conclusion, the increased genetic drift caused by smaller N_e of success-selected lines can produce $\mathbf{P_{S<F}}$ and $\mathbf{D}\boldsymbol{H}_\mathbf{e}$ values comparable to our observed values, but such low N_e values inflate pairwise F_{ST} values among success-selected lines (i.e. substantially increase $\mathbf{D}\boldsymbol{F}_\mathbf{ST}$) to an extent not observed in our data. Thus, the results from these simulations strongly suggest that sexual selection has caused the reduction in H_e in success-selected lines, thereby confirming the main premise of the lek paradox.

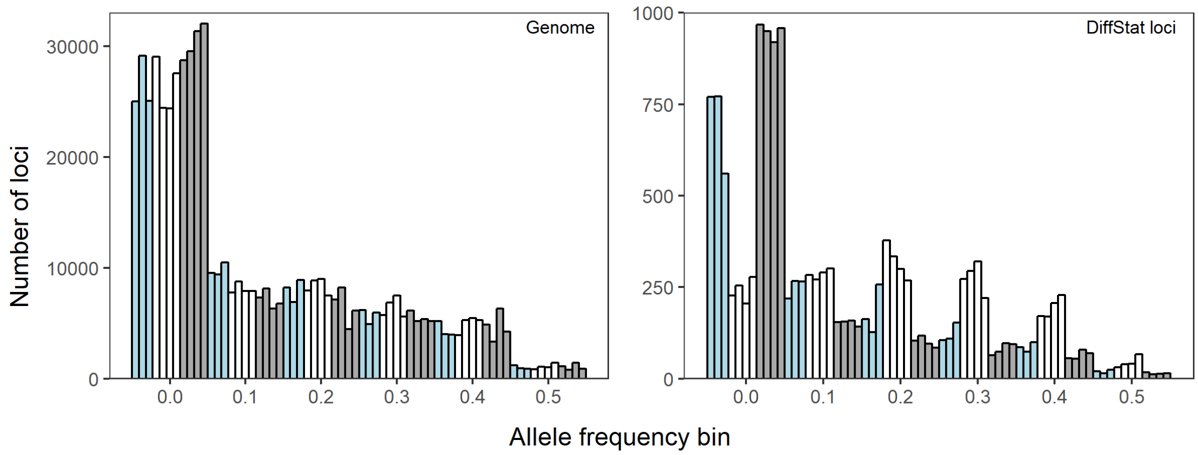

Supplementary Figure 1 | Allele frequency spectra of control (blue), failure-selected (white), and success-selected (grey) lines for all loci (left) and *DiffStat* loci (right). Success-selected lines have substantially more fixed alleles at *DiffStat* loci than failure-selected lines, indicating that selection eroded genetic variation.

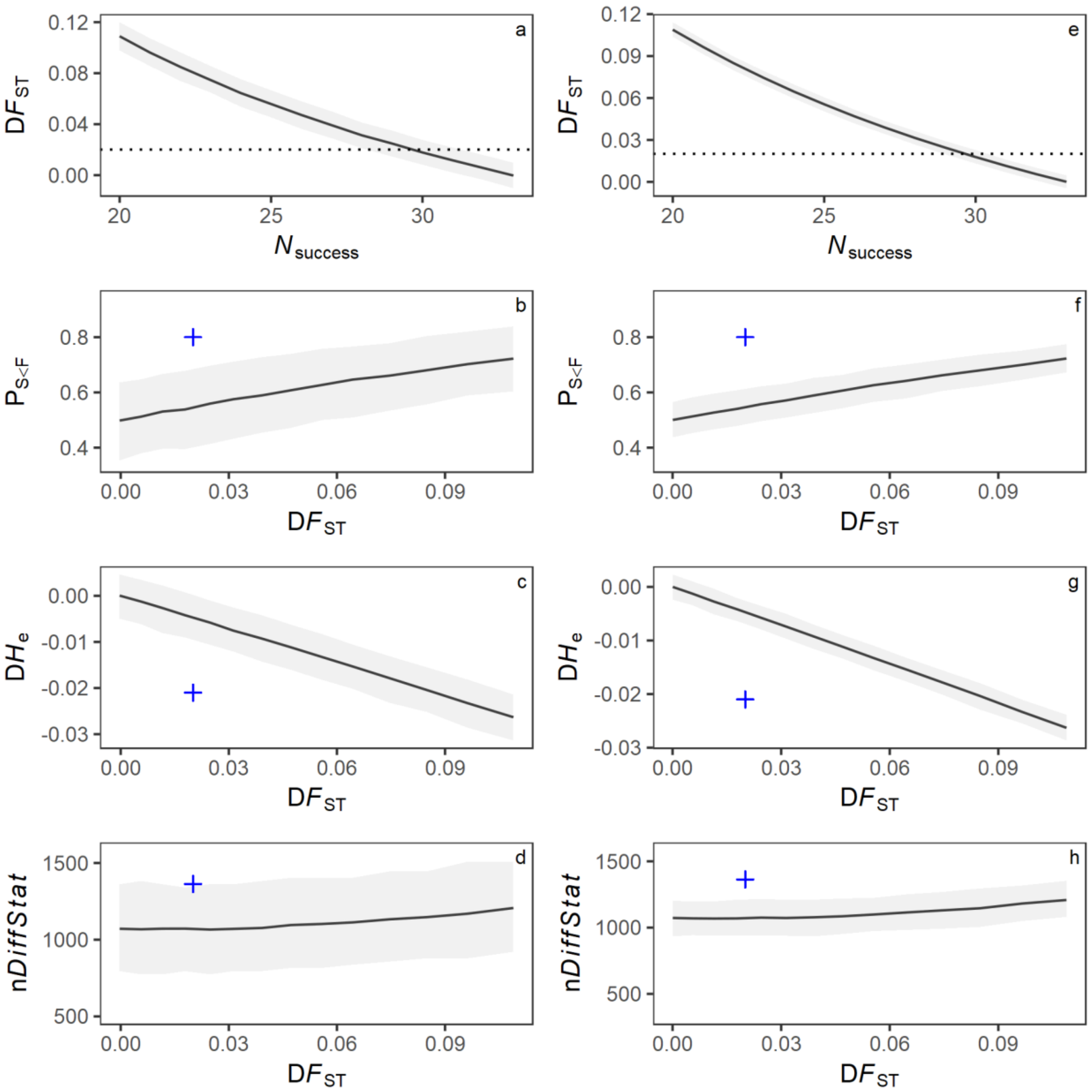

Supplementary Figure 2 | Simulation results from 14 generations of genetic drift. Panels represent: (a, e) the size of the success-selected populations ($N_{success}$) against the difference in among line F_{ST} values between success- and failure-selected lines (DF_{ST}). The population size of the failure-selected lines was held constant at $N_{failure} = 33$. The DF_{ST} values substantially increase as $N_{success}$ decreases; (b, f) the relationship between DF_{ST} and the proportion of *DiffStat* loci where the expected heterozygosity (H_e) is lower in success-selected lines ($P_{S<F}$); (c, g) the relationship between DF_{ST} and the difference in H_e values between success- and failure-selected lines (DH_e); (d, h) the relationship between the number of *DiffStat* loci (n*DiffStat*) and DF_{ST}. 2,640 (a-d) and 12,424 (e-h) loci were used in the simulations. Lines represent the mean values across 1,000 simulations; shaded areas are 95% confidence intervals. Blue crosses (and dashed lines in a, e) represent the observed values, which all fall outside of the 95% confidence intervals. See methods and Supplementary Discussion for full details of the simulations.

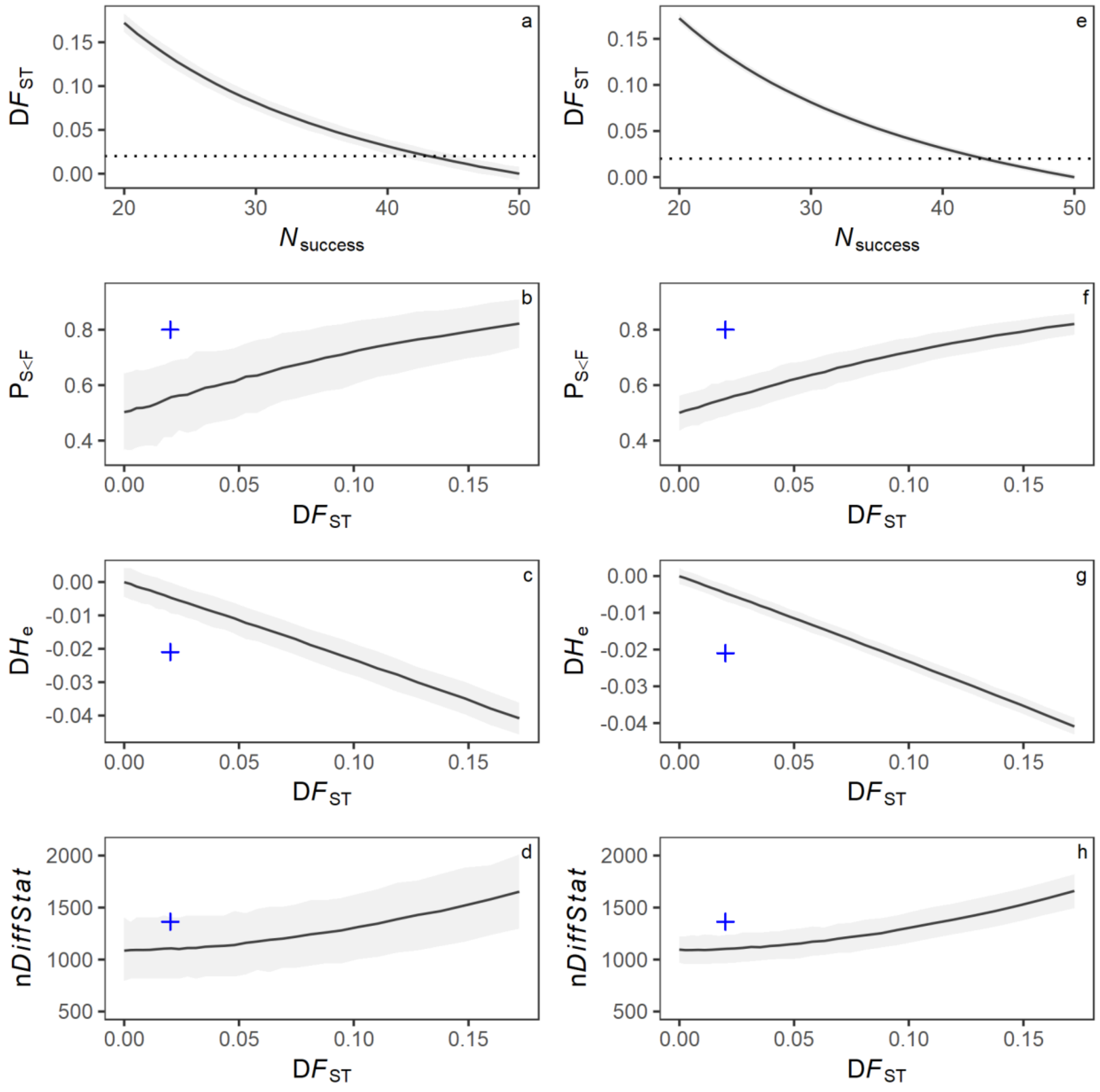

Supplementary Figure 3 | Simulation results from 14 generations of genetic drift. Panels represent: (a, e) the size of the success-selected populations (N_{success}) against the difference in among line F_{ST} values between success- and failure-selected lines (DF_{ST}). The population size of the failure-selected lines was held constant at $N_{\text{failure}} = 50$. The DF_{ST} values substantially increase as N_{success} decreases; (b, f) the relationship between DF_{ST} and the proportion of *DiffStat* loci where the expected heterozygosity (H_e) is lower in success-selected lines ($P_{S<F}$); (c, g) the relationship between DF_{ST} and the difference in H_e values between success- and failure-selected lines (DH_e); (d, h) the relationship between the number of *DiffStat* loci (n*DiffStat*) and DF_{ST}. 2,640 (a-d) and 12,424 (e-h) loci were used in the simulations. Lines represent the mean values across 1,000 simulations; shaded areas are 95% confidence intervals. Blue crosses (and dashed lines in a, e) represent the observed values, which largely fall outside of the 95% confidence intervals. See methods and Supplementary Discussion for full details of the simulations.

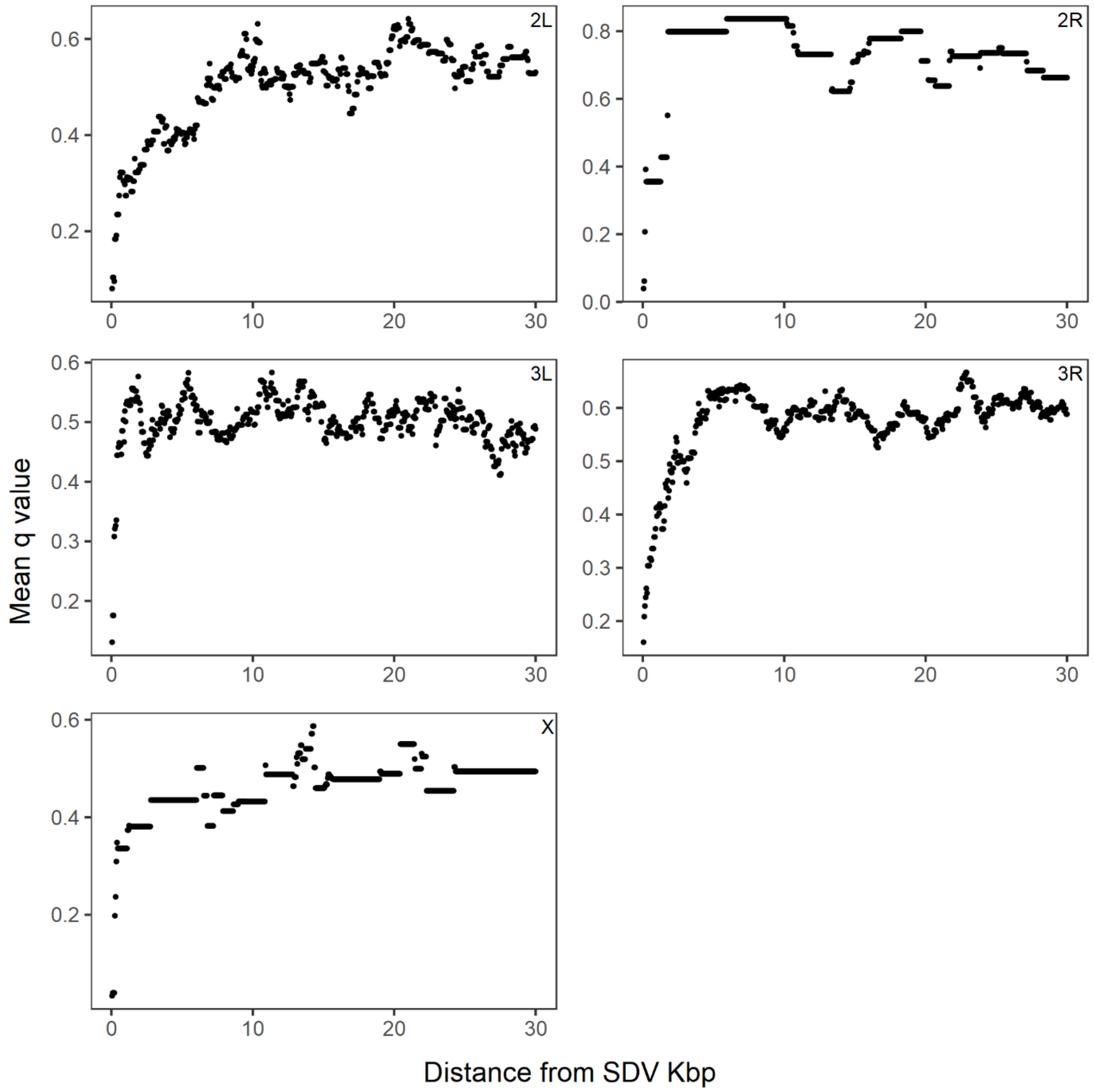

Supplementary Figure 4 | Estimated extent of linkage disequilibrium (LD) for the five chromosome arms. On the x-axis is the distance (in kbps) from a significantly diverged variant (SDV) and on the y-axis is the average *q* value at that distance. Mean *q* values were calculated in 50 bp, non-overlapping sliding windows. Loci linked to SDVs have lower *q* values indicating that they are in LD; our estimate of when LD breaks down is when the *q* values plateau. LD varies across chromosome arms, but does not exceed 10 kbps.

Supplementary Table 1 | Median expected heterozygosity in 50 kb sliding windows with 10 kb step sizes across each of the five major chromosome arms. The last three columns are the means for each selection regime. C = control, F = failure-selected, S = success-selected.

Chrom	*n*	C1	C2	C3	F1	F2	F3	F4	S1	S2	S3	S4	C	F	S
**2L**	2280	0.17	0.16	0.20	0.12	0.15	0.22	0.21	0.21	0.17	0.16	0.13	0.17	0.18	0.17
**2R**	2406	0.20	0.17	0.14	0.14	0.18	0.21	0.16	0.20	0.21	0.12	0.18	0.17	0.17	0.18
**3L**	2656	0.19	0.16	0.19	0.16	0.19	0.18	0.14	0.12	0.15	0.15	0.14	0.18	0.17	0.14
**3R**	2999	0.19	0.12	0.19	0.20	0.23	0.21	0.17	0.14	0.10	0.18	0.13	0.17	0.20	0.13
**X**	2140	0.15	0.12	0.15	0.14	0.14	0.07	0.17	0.12	0.15	0.14	0.13	0.14	0.13	0.13
**Total**	12481	0.18	0.15	0.18	0.16	0.18	0.19	0.17	0.16	0.16	0.15	0.14	**0.17**	**0.17**	**0.15**

Supplementary Table 2 | Median expected heterozygosity for windows with >1 SDV. The last three columns are the means for each selection regime. C = control, F = failure-selected, S = success-selected.

**Chrom**	***n***	**C1**	**C2**	**C3**	**F1**	**F2**	**F3**	**F4**	**S1**	**S2**	**S3**	**S4**	**C**	**F**	**S**
**2L**	12	0.01	0.21	0.25	0.27	0.25	0.21	0.21	0.11	0.03	0.04	0.04	0.16	0.24	0.06
**2R**	5	0.36	0.13	0.07	0.19	0.24	0.29	0.25	0.05	0.23	0.05	0.08	0.19	0.24	0.10
**3L**	13	0.07	0.13	0.21	0.29	0.25	0.28	0.13	0.06	0.12	0.08	0.12	0.13	0.24	0.10
**3R**	22	0.19	0.10	0.15	0.18	0.23	0.23	0.24	0.14	0.00	0.19	0.04	0.15	0.22	0.09
**X**	5	0.09	0.13	0.14	0.15	0.17	0.20	0.20	0.10	0.16	0.04	0.22	0.12	0.18	0.13
**Total**	57	0.11	0.12	0.17	0.21	0.23	0.25	0.20	0.10	0.06	0.10	0.08	**0.13**	**0.22**	**0.08**

Supplementary Table 3 | Mapping statistics for all 11 lines. Number of reads indicates the total number of forward and reverse reads.

Sample	Reads	Mapped	Mapped %	Mean depth	Haploid variants
Control 1	44,944,410	44,261,349	98.48%	30.5	652,199
Control 2	43,096,592	41,338,434	95.92%	28.4	682,394
Control 3	44,339,040	42,768,210	96.46%	29.4	655,656
Failure 1	44,616,266	44,079,965	98.80%	30.3	673,167
Failure 2	43,780,810	39,683,978	90.64%	27.3	656,037
Failure 3	43,114,444	41,244,751	95.66%	28.5	671,936
Failure 4	46,483,170	44,570,062	95.88%	30.4	690,576
Success 1	44,538,442	42,565,945	95.57%	29.4	656,921
Success 2	45,769,226	43,444,656	94.92%	30.0	685,515
Success 3	43,922,024	43,510,469	99.06%	30.2	683,394
Success 4	45,070,606	43,388,427	96.27%	29.8	690,795
